# Recommendations for Addressing In-Hospital Substance Use From a National Delphi Consensus Process

**DOI:** 10.1001/jamanetworkopen.2025.28703

**Published:** 2025-08-25

**Authors:** Joseph H. Donroe, Susan L. Calcaterra, Caty Simon, Melissa B. Weimer, Zina Huxley-Reicher, Lisa B. Puglisi, Kristine Torres-Lockhart, Orman Trent Hall, James Bhandary-Alexander, John Encandela, Marlene Martin

**Affiliations:** 1 Department of Internal Medicine, Yale School of Medicine, New Haven, Connecticut; 2 Program in Addiction Medicine, Section of General Internal Medicine, Yale School of Medicine, New Haven, Connecticut; 3 Department of Medicine, Divisions of General Internal Medicine and Hospital Medicine, University of Colorado, Aurora; 4 National Survivors Union, Greensboro, North Carolina; 5 Department of Social and Behavioral Sciences, Yale School of Public Health, New Haven, Connecticut; 6 Yale School of Public Health, New Haven, Connecticut; 7 Department of Medicine, NYC Health + Hospitals/Woodhull, New York, New York; 8 Department of Medicine, Division of General Internal Medicine, Montefiore Medical Center/Albert Einstein College of Medicine, Bronx, New York; 9 Department of Psychiatry and Behavioral Health, Ohio State University Wexner Medical Center, Columbus; 10 Solomon Center for Health Law and Policy at Yale Law School, New Haven, Connecticut; 11 Center for Medical Education, Yale School of Medicine, New Haven, Connecticut; 12 Department of Medicine, San Francisco General Hospital, San Francisco, California; 13 Department of Medicine, University of California, San Francisco

## Abstract

**Importance:**

When people who use drugs are hospitalized, they may use nonprescribed substances, including opioids, stimulants, and cannabis. Most hospitals do not have standardized approaches to address such in-hospital substance use, which may lead to harm and bias.

**Objective:**

To develop best-practice recommendations to address in-hospital substance use.

**Design, Setting, and Participants:**

This survey study using 3 rounds of a Delphi process included clinicians and people with lived or living experience (LLE) of substance use disorders. Eligible clinician experts were physicians, advanced practice clinicians, nurses, and social workers who had worked on a hospital-based addiction consultation team for at least 4 weeks in the past year. Eligible experts with LLE self-identified as meeting diagnostic criteria for substance use disorder and experiencing a hospitalization in the prior 10 years. Participants were recruited by email through author contacts and snowball sampling and completed online surveys from February 27 to June 25, 2024.

**Main Outcomes and Measures:**

The main outcome was development of consensus recommendations to address in-hospital substance use. Using a Delphi process, addiction experts identified best-practice recommendations through 3 rounds of surveys. The final survey included 8 categories of recommendations: at the time of hospital admission, once in-hospital substance use is suspected, management of substances and substance use supplies if found, personnel involved in the response, clinical management, documentation, expected outcomes of the response, and systems-level approaches. A content analysis of the free-text comments associated with recommendations meeting consensus for “sometimes implement” or “rarely implement” was performed to better understand when these recommendations should be implemented.

**Results:**

Of 87 experts contacted, 38 participated (response rate, 44%), including 21 clinicians without LLE of SUD (55%), 10 nonclinicians with LLE (26%), and 7 clinicians with LLE (18%). Mean (SD) participant age was 40.3 (10.0) years, and 24 (63%) were cisgender female. Experts identified 84 consensus recommendations, including 49 (58%) that should always be implemented, 4 (5%) that should never be implemented, and 31 (37%) that should be considered depending on the patient and circumstances. Five key concepts were identified in the content analysis of comments related to recommendations with group consensus to sometimes implement or rarely implement: patient autonomy, evidence-based care, feasibility, impact, and harms and unintended consequences.

**Conclusions and Relevance:**

In this survey study, an expert panel identified best-practice recommendations to address in-hospital substance use, including recommendations to implement and avoid. These recommendations can inform local responses, including hospital policies, to standardize responses to in-hospital substance use in a patient-centered and evidence-based way.

## Key Points

**Question:**

What are best practices for addressing in-hospital substance use?

**Findings:**

In this survey study using a 3-round Delphi consensus process, a panel of 38 addiction experts developed 84 consensus recommendations to address in-hospital substance use.

**Meaning:**

This comprehensive set of patient-centered and consensus-based recommendations can inform local responses, including policies, to address in-hospital substance use.

## Teaser

This survey study including substance use disorder experts used a Delphi consensus process to develop best-practice recommendations for addressing patients’ in-hospital substance use.

## Introduction

In the US and Canada, the prevalence of in-hospital substance use among people with a substance use disorder (SUD) is as high as 30% to 44%.^1,2,3,4,5^ When people who use drugs are hospitalized, they may use substances to treat withdrawal, pain, and anxiety or mitigate cravings and boredom.^3,6,7,8,9^ There are myriad reasons why people who use drugs may not receive adequate addiction treatment during a hospitalization, including stigma and lack of health care worker education and training.^10,11,12,13,14,15,16^

Health care worker responses to ongoing or suspected in-hospital substance use can cause harm to patients and health care workers and generate systems-level concerns. For patients, harms include an increased risk of patient-directed discharges, legal consequences when substance use is reported to the police, child protective service involvement for pregnant and parenting patients, weakening of the patient–health care worker relationship, financial consequences when substances and substance use supplies are confiscated, a reluctance for people to engage in health care in the future, and other harmful outcomes, including adverse drug interactions, unintentional overdose, riskier substance use behaviors, infection, altered mental status, and death.^1,5,7,17,18^ For health care workers, in-hospital substance use can engender mistrust, lead to further stigmatization of people who use drugs, and cause stress regarding licensing and personal safety concerns (eg, needle stick injuries).^6,7^ At the system level, hospitals may worry about regulatory or liability issues.^19^

Addressing ongoing or suspected in-hospital substance use can be standardized through a hospital policy informed by best-practice recommendations.^7^ A patient-centered thoughtfully informed policy can minimize patient, health care worker, and hospital harms and address and mitigate reasons for in-hospital substance use through evidence-based practices that respect patient autonomy and strengthen the patient–health care worker relationship. However, few hospitals have such policies. One study of US hospitals with Accreditation Council for Graduate Medical Education fellowship programs found that only 22% of respondents reported awareness of a hospital policy to address in-hospital substance use.^20^

To our knowledge, the literature to date on addressing in-hospital substance use includes single-site recommendations.^5,7^ These emphasize remaining patient-centered, avoiding punitive measures, educating health care workers on evidence-based SUD care, treating pain and withdrawal, and developing hospital policies to address in-hospital substance use.^7,21,22,23,24^ Additionally, there are no national standards on addressing in-hospital substance use, including from the Joint Commission, other than recommendations to assess and treat SUD.^25,26^ Given the scarce guidance for best practices to address in-hospital substance use and inform local responses and hospital policy, we aimed to develop consensus recommendations.

## Methods

In this survey study, we used a Delphi process to develop in-hospital substance use recommendations. The Delphi process is a highly structured group interaction among highly informed subject matter experts with professional and/or lived experience.^27^ The process facilitates consensus building and is particularly useful when empirical evidence is lacking.^28^ We chose the Delphi process over other consensus development strategies because it is anonymous and avoids the potential for group power dynamics to influence opinions, involves multiple rounds for participants to consider their recommendations, and provides the flexibility for respondents to participate according to their own schedules.^29^ We followed the Standards for Quality Improvement Reporting Excellence (SQUIRE) guidelines when applicable. We retrospectively confirmed alignment with the Accurate Consensus Reporting Document (ACCORD) checklist, as this was published after we designed our study.^30^ The study was approved by the institutional review boards of Yale University and the University of California, San Francisco. All experts provided written consent to participate and received a $50 prepaid debit card on final survey completion.

### Recruiting the Expert Panel

There is no established cutoff for sample size in a Delphi process, although a panel of 15 to 30 participants from the same discipline (or 5 to 10 per discipline if the panel is heterogenous) can be effective.^31^ We recruited experts with a goal of having an equal distribution of physician and nonphysician clinicians and people with lived or living experience (LLE) of SUD. We only included experts who agreed that a hospital policy should exist to address in-hospital substance use or suspected use of substances. We initially recruited clinicians and people with LLE through author group contacts, and we then used snowball sampling to recruit from contacts of those who responded to the invitation email. Snowball sampling is effective for recruiting participants who might otherwise be missed, such as those facing stigma, by working with social networks to assist with recruitment.^32^

Eligible clinicians were physicians, advanced practice practitioners, social workers, or nurses who practiced hospital-based addiction care for at least 4 weeks in the past year. We included this range of specialties and time frame to minimize bias from a single profession and to ensure recent experience with in-hospital care of patients with SUD. People with LLE were eligible to participate if they self-identified as having an SUD (excluding only alcohol and/or tobacco use disorders) and (1) had a hospitalization within the past 10 years if they also had an active role advocating for people with LLE or worked in health care or (2) had a hospitalization within the past 5 years if they did not. We selected this time frame to balance recency of hospitalization with challenges in recruitment. All experts self-selected gender and racial and ethnic identity using predefined options in the survey. Race and ethnicity were included in the analysis for a better understanding of the representativeness of our study sample of experts.

### Developing the Survey

We began meeting in October 2023 to iteratively develop the survey. The initial survey contained 70 recommendations for addressing in-hospital substance use and was informed by literature review, data collected from a prior study of in-hospital substance use policies,^20^ and existing policies from select hospitals. Recommendations were initially divided into 7 categories: recommendations at the time of admission (n = 13); investigation once substance use is suspected (n = 9); management of substances and substance use supplies if found (n = 6); personnel involved in the response (n = 11); clinical management once substance use is suspected or confirmed (n = 12); documentation (n = 5); and outcomes from the response to in-hospital substance use (n = 14). We piloted the survey among the author group, and based on the responses, we added 6 new recommendations to the final round 1 survey (eAppendix in Supplement 1) for a total of 76 recommendations.

### Delphi Process

Experts participated in 3 rounds of surveys (eFigure 1 in Supplement 1) between February 27 and June 25, 2024, and prioritized the recommendations using the following categories: “always implement,” “sometimes implement,” “rarely implement,” “never implement,” and “undecided.” A priori, we determined consensus as at least 80% agreement among experts, indeterminate consensus as 60% to less than 80% agreement, and lacking consensus as less than 60% agreement.^33^ In every round, experts could comment on the language used for each recommendation, elaborate on nuances about when recommendations should or should not be implemented, and offer new recommendations. After round 1, we advanced new recommendations and recommendations in the indeterminate consensus range to the subsequent round, and we did the same after round 2. Recommendations lacking consensus were not included in subsequent rounds of surveys. Surveys in rounds 2 and 3 contained aggregate consensus data from the prior round. We predetermined that the Delphi process would end after 3 rounds of expert responses.

### Statistical Analysis

We administered the surveys and collected data using a secure web-based platform (Qualtrics; Qualtrics Ltd). We exported survey data to IBM SPSS, version 28.0.1.1 (14) (IBM Corp), for descriptive analysis and between-group comparisons. For the category of “sometimes implement,” we considered consensus to be met if “sometimes implement” alone or the sum of “always implement” and “sometimes implement” had 80% agreement or more. For the category of “rarely implement,” we considered consensus to be met if “rarely implement” alone or the sum of “never implement” and “rarely implement” had agreement of 80% or more. We used the Mann-Whitney *U* test to compare the distribution of responses between clinicians and people with LLE for each recommendation and set the level of significance at 2-sided *P* <.05. For this analysis, we included people with LLE who were also clinicians in the group of people with LLE.

We performed a content analysis of the free-text comments associated with recommendations meeting consensus for “sometimes implement” or “rarely implement” to better understand when these recommendations should be implemented.^34,35^ We created a priori codes based on our clinical expertise and review of the literature. Two authors trained in qualitative methods (J.H.D., Z.H.R.) independently reviewed the relevant free-text responses and applied a priori codes. They also independently added new codes as ideas surfaced in the free-text responses. They then reviewed and discussed the codes until they reached consensus. Three authors (J.H.D., Z.H.R., M.M.) then reviewed the codes, agreed on the key concepts that emerged, and quantified the number of responses per concept.

## Results

We contacted 87 potential participants (55 clinicians and 32 people with LLE) by email. Twenty-five of the clinicians (45%) and 13 of the people with LLE (41%) responded and met inclusion criteria, for a total of 38 experts included (response rate, 44%). Of the 38 experts, 21 (55%) identified as clinicians without LLE of SUD, 10 (26%) as nonclinicians with LLE, and 7 (18%) as clinicians with LLE. Mean (SD) age was 40.3 (10.0) years; 24 participants (63%) identified as cisgender female, 12 (32%) as cisgender male, 1 (3%) as nonbinary, and 1 (3%) as transgender male. One participant (3%) identified as American Indian, 7 (18%) as Asian, 1 (3%) as Middle Eastern, 1 (3%) as Roma, and 33 (87%) as White (race and ethnicity categories were not mutually exclusive). Ten of the people with LLE (77%) had a role advocating for people who use drugs. Of the 13 nonclinicians with LLE, 6 (46%) were hospitalized within the past year, 4 (31%) within 1 to 3 years, and 3 (23%) within 3 to 5 years. Among the 25 clinicians working on an addiction consultation service, 10 (40%) spent 4 to 10 weeks, 6 (24%) spent 10 to 20 weeks, and 9 (36%) spent more than 20 weeks in the past year caring for people with SUD on an addiction consultation team. Their median number of years of experience in addiction was 6.0 (range, 1-25 years). All physicians and 2 of the 7 advanced practice clinicians (29%) reported board certification in addiction medicine or addiction psychiatry. Table 1 has further details regarding participants.

**Table 1.** Demographics and Characteristics of Expert Panel Participants With and Without LLE of SUD

Characteristic	Round 1 experts (N = 38)^a^
Total participants	
Nonclinicians with LLE	10 (26)
Clinicians with LLE	7 (18)
Clinicians without LLE	21 (55)
Clinician type, No./total No. (%)	
Physician	13/28 (46)
Advanced practice clinician	7/28 (25)
Nurse	3/28 (11)
Social worker	5/28 (18)
Age, mean (SD), y	40.3 (10.0)
Gender	
Cisgender female	24 (63)
Cisgender male	12 (32)
Nonbinary	1 (3)
Transgender male	1 (3)
Racial and ethnic identity^b^	
American Indian	1 (3)
Asian	7 (18)
Middle Eastern	1 (3)
Roma	1 (3)
White	33 (87)
US region of hospital or residence^c^	
Northeast	10 (26)
South	8 (21)
Midwest	4 (11)
West	16 (42)
Clinician’s hospital setting, No./total No. (%)^b^	
Urban	24/25 (96)
Academic	21/25 (84)
Community	4/25 (16)
Nonprofit	8/25 (32)
Beds, No.	
<500	9/25 (36)
500-1000	12/25 (48)
>1000	4/25 (16)
Addiction consultation team	24/25 (96)
Identities of people with LLE, No./total No. (%)^b^	
Lived experience	7/13 (54)
Living experience	7/13 (54)
In recovery	8/13 (62)
>12 mo In recovery	5/13 (38)
Advocacy role	10/13 (77)
SUD	
Opioid	10/13 (77)
Alcohol	5/13 (38)
Stimulant	8/13 (62)
Cannabis	4/13 (31)
Phencyclidine	1/13 (8)
Sedative	5/13 (38)
Hallucinogen	2/13 (15)
Inhalant	1/13 (8)
Clinician specialty, No./total No. (%)	
Addiction	7/25 (28)
Internal medicine	1/25 (4)
Internal medicine, addiction	11/25 (44)
Family medicine	1/25 (4)
Family medicine, addiction	1/25 (4)
Psychiatry, addiction	2/25 (8)
Not applicable	2/25 (8)

Abbreviations: LLE, lived or living experience; SUD, substance use disorder.

^a^ Data are presented as number (percentage) of participants unless otherwise indicated. For clinicians, the denominator is 25 when limited to those responding to the initial clinician survey and 28 when including those from the PWLLE survey who also identified as clinicians.

^b^ Categories are not mutually exclusive.

^c^ Hospital location was used for clinicians and residence location for people with LLE.

All 38 experts participated in the round 1 survey, and they reached consensus on 31 of 76 recommendations (41%). In addition to 26 new recommendations suggested by experts, we moved 37 indeterminate recommendations to round 2. Based on round 1 feedback, we added an eighth category to the round 2 survey, “Systems-level approach to addressing in-hospital substance use,” containing 5 new recommendations. In round 2, 35 experts (92%) found consensus on 31 of 63 additional recommendations (49%). We moved the 2 recommendations newly suggested by round 2 experts and 23 indeterminate recommendations to round 3. In round 3, 35 experts (92%) reached consensus on 22 of the 25 recommendations (88%). The flow of recommendations through each round, including recommendations that did not meet consensus, is depicted in eFigure 2 in Supplement 1.

Of the 84 recommendations that reached consensus (Table 2), experts categorized 49 (58%) as “always implement,” 4 (5%) as “never implement,” and 31 (37%) as recommendations that should be considered depending on the patient and circumstances, which included 22 recommendations (26%) categorized as “sometimes implement” and 9 (11%) as “rarely implement.” Twenty recommendations did not reach consensus (eTable in Supplement 1). Clinicians and people with LLE had statistically significant differences in the distribution of responses among 10 recommendations that reached consensus and 6 that did not reach consensus (Table 3). In the content analysis of comments related to recommendations with consensus to sometimes implement or rarely implement, we identified 5 key concepts, focused on patient autonomy, evidence-based care, feasibility, impact, and harms and unintended consequences (Table 4).

**Table 2.** Consensus Recommendations by Category

Recommendation^a^	Experts in agreement, No./total No. (%)
**All patients at time of admission**	
Always implement	
Staff should assess a patient’s risk of withdrawal from substances	34/38 (89)
Medication for substance use disorder treatment should be offered if a patient meets criteria	38/38 (100)
The patient’s pain management plan should be discussed, if applicable	38/38 (100)
The patient’s withdrawal management plan should be discussed, if applicable	36/38 (95)
Patients should be offered the opportunity to discreetly dispose of substances or substance use supplies	32/38 (84)
Patients should be made aware of the hospital’s substance use policies	37/38 (97)
Patients should be made aware of when their right to privacy may be superseded by the hospital’s obligation to provide safe medical care	36/38 (95)
Patients should be made aware of why it is unsafe to use substances during a hospitalization	32/38 (84)
Patients should be made aware of visitor expectations	34/38 (89)
Patients with tobacco use disorder should be offered nicotine replacement therapy	37/38 (97)
Patients should be offered the opportunity to discreetly store substances or substance use supplies with their belongings until time of discharge	30/35 (86)
Patients should be asked about their goals for their substance use disorder (if one is present)	31/35 (89)
Patients with substance use disorders should be asked if they have ever needed to leave the hospital due to uncontrolled withdrawal or cravings or if they’re worried that might occur	29/35 (83)
Staff should assess all patients for substance use disorders	30/35 (88)
Patients should be provided a handout describing hospital resources for people with substance use disorders	30/36 (86)
Patients should be provided a handout outlining policies around inpatient substance use	31/35 (88)
Patients should be provided a handout describing methods to discretely dispose of substances	32/35 (91)
Patients should be provided a handout describing the approach to withdrawal management	32/35 (91)
Patients should be provided a handout describing the approach to pain management	30/35 (86)
If withdrawal is poorly controlled, patients should be temporarily offered short-acting opioid medications in addition to methadone or buprenorphine	28/35 (80)
Sometimes implement	
Patients with tobacco use disorder should be allowed to smoke or vape in designated areas	31/35 (89)
Patients on oxygen should not be allowed to smoke or vape in designated areas	29/35 (83)
If pain is poorly controlled, patients should be offered short-acting opioid medications during the hospitalization	35/35 (100)
Hospital policy around inpatient substance use should be discussed by a peer advocate	31/35 (99)
Rarely implement	
Patients with a history of substance use disorder should be searched	33/35 (94)
**Investigation once substance use is suspected**	
Always implement	
Patients’ consent should be obtained before toxicology is performed, whenever possible	34/38 (89)
Patients should be informed about why the toxicology test is being performed	36/38 (95)
Patients should be informed about how the results will be used in their care	36/38 (95)
Patient consent should be obtained prior to a search of room and personal belongings	34/38 (89)
When available, the patient should be offered a peer advocate to assist with patient-staff interactions	35/38 (92)
Staff should explore with the patient tools and resources they would find helpful to tolerate the hospital stay	34/35 (97)
Sometimes implement	
Assessment of patient decision-making capacity should be performed (a clinician would evaluate the patient’s ability to make their own medical decisions in that moment)	30/35 (86)
Patients should be asked if they are actively using substances in the hospital	30/35 (86)
Screening toxicology should be followed by confirmatory testing	29/35 (83)
Staff should increase the frequency of patient check-ins	31/35 (89)
If considered necessary, patients should be told that a search will be conducted, while providing specific reasons	30/35 (86)
Rarely implement	
The patient’s room and belongings should be searched by hospital security	32/35 (91)
**Management of substances and substance use supplies if found**	
Always implement	
Patient confidentiality should be maintained (evidence linking the patient to any illegal substances is avoided)	34/38 (89)
Patients should consent to the disposal of substances	30/35 (86)
Patients should consent to the disposal of substance use supplies	28/35 (80)
Sometimes implement	
Substance use supplies should be stored in a locked space during hospitalization	29/34 (85)
Substance use supplies should be returned to patients at time of discharge, if they desire	32/34 (94)
Substances should be returned to patients at time of discharge, if they desire	33/35 (94)
Never implement	
Suspected illegal substances should be given to local law enforcement	35/38 (92)
**Personnel involved**	
Always implement	
The patient’s primary medical or surgical team should be involved	29/35 (83)
The floor nursing staff should be involved	28/35 (80)
Sometimes implement	
Peer advocates should be involved	32/34 (94)
An addiction specialist should be involved	32/24 (94)
A multidisciplinary group should be created to address inpatient substance use	29/34 (85)
A psychiatry consultant or behavioral health team should be involved	29/34 (85)
Patient advocates (patient relations) should be involved	29/34 (85)
A social worker should be involved	28/34 (82)
A dedicated team of addiction-trained staff should respond to suspected inpatient substance use	33/35 (94)
Rarely implement	
Hospital security personnel should be involved	32/34 (94)
Never implement	
Local law enforcement should be involved	32/38 (84)
The patient’s parole or probation officer should be alerted (if applicable)	35/38 (92)
**Clinical management**	
Always implement	
The patient’s pain management plan should be assessed or reassessed	31/38 (82)
The patient’s withdrawal management plan should be assessed or reassessed	33/38 (87)
The patient’s reasons for use (craving, stress, anxiety) should be assessed or reassessed	31/38 (82)
Medications for symptom management should be offered	36/38 (95)
Medication for addiction treatment should be offered if patient meets criteria	37/38 (97)
Addiction medication dosage should be increased if appropriate and patient desires	34/38 (89)
Available harm reduction modalities should be discussed	37/38 (97)
The patient should be reminded of the hospital’s policy, including next steps should substance use continue	32/38 (84)
The patient should be provided a list of resources and asked what resources they would like support from	33/34 (97)
Sometimes implement	
Patients should be offered psychosocial treatment groups during the hospitalization (if available)	33/34 (97)
A multidisciplinary meeting should be held to develop a plan to manage suspected or confirmed substance use during the hospitalization	32/34 (94)
**Documentation**	
Sometimes implement	
If documenting inpatient substance use in the medical record, implement standardized documentation templates	32/35 (91)
Rarely implement	
An alert should be added to the patient’s medical record identifying them as someone who used substances during their hospitalization	29/34 (85)
**Outcomes from the response to inpatient substance use**	
Always implement	
Patients who self-direct their discharge should be connected to outpatient addiction care, if they desire	35/38 (92)
Patients who self-direct their discharge should be prescribed medications for addiction treatment, if they desire	36/38 (95)
Patients who self-direct their discharge should be provided with harm reduction modalities (ie, naloxone, syringes, skin prep), if they desire	37/38 (97)
A pathway to reestablish lost privileges (eg, visitors, spacing out frequency of staff visits) during the hospitalization should be available	32/38 (84)
Rarely implement	
Patients should be restricted to their room	34/34 (100)
Furniture with drawers and any personal containers where syringes, drugs, or alcohol could be stored should be removed from the patient’s room	29/34 (88)
Restriction of ability to leave the medical ward should be implemented	30/34 (82)
A safety attendant (in person or remotely) should be ordered to monitor the patient	28/34 (82)
If patient visitors are not restricted, all visitors should be searched prior to visitation	33/35 (94)
Never implement	
Patients should be instructed to self-direct their discharge (“discharge against medical advice”) from the hospital if they refuse to comply with hospital policy	28/35 (80)
**Systems-level approach to addressing inpatient substance use**	
Always implement	
Hospitals should provide up-to-date treatment of substance use disorders	33/34 (97)
Hospitals should provide staff with ongoing education on harm reduction practices	34/34 (100)
Hospital policy addressing inpatient substance use should be nonstigmatizing	34/34 (100)
Hospital policy addressing inpatient substance use should serve to improve partnership with patients	33/34 (97)
Hospitals should have a protocolized response to suspected inpatient use	28/35 (80)

^a^ Recommendation wording is presented verbatim from the survey.

**Table 3.** Recommendations With Significant Differences in Responses Between Clinicians and People With LLE

Recommendation	Experts, No./total No. (%)					*P* value
	Always	Sometimes	Rarely	Never	Undecided	
**Round 1**						
Staff should assess patients for substance use disorders at the time of admission^a^						
Clinicians	19/21 (90)	2/21 (10)	0	0	0	.02
People with LLE	8/17 (47)	7/17 (41)	0	0	2/17 (12)	
Patients should be asked if they are actively using substances in the hospital once substance use is suspected^a^						
Clinicians	17/21 (81)	4/21 (19)	0	0	0	.002
People with LLE	5/17 (29)	7/17 (41)	2/17 (12)	1/17 (6)	2/12 (12)	
The patient’s primary medical or surgical team should be involved in a response to suspected or confirmed in-hospital substance use^a^						
Clinicians	16/21 (76)	5/21 (24)	0	0	0	.01
People with LLE	6/17 (35)	7/17 (41)	1/17 (6)	1/17 (6)	2/17 (12)	
The floor nursing staff should be involved in a response to suspected or confirmed in-hospital substance use^a^						
Clinicians	15/21 (71)	4/21 (19)	0	1/21 (5)	1/21 (5)	.04
People with LLE	6/17 (35)	4/17 (24)	3/17 (18)	2/17 (12)	2/17 (12)	
Investigation into suspected substance use should be documented in the patient’s medical record						
Clinicians	5/21 (24)	7/21 (33)	4/21 (19)	3/21 (14)	2/21 (10)	.02
People with LLE	1/17 (6)	1/17 (6)	5/17 (29)	9/17 (53)	1/17 (6)	
Reasons for suspecting substance use during the hospitalization should be documented in the patient’s medical record						
Clinicians	6/21 (29)	8/21 (38)	4/21 (19)	1/21 (5)	2/21 (10)	.03
People with LLE	2/17 (12)	3/17 (18)	3/17 (18)	8/17 (47)	1/17 (6)	
The management of suspected or confirmed substance use in the hospital should be documented in the patient’s medical record						
Clinicians	9/21 (43)	9/21 (43)	3/21 (14)	0	0	.007
People with LLE	4/17 (24)	2/17 (12)	5/17 (29)	5/17 (29)	1/17 (6)	
In the response to substance use in the hospital, if visitors are allowed, all visitors should be searched prior to visitation^a^						
Clinicians	1/21 (5)	4/21 (19)	5/21 (24)	9/21 (43)	2/21 (10)	.04
People with LLE	0	0	2/17 (12)	12/17 (71)	3/17 (18)	
In the response to substance use in the hospital, the patient’s room should be relocated to a more easily observable location						
Clinicians	1/21 (5)	7/21 (33)	10/21 (48)	3/21 (14)	0	.01
People with LLE	1/17 (6)	1/17 (6)	6/17 (35)	6/17 (35)	3/17 (18)	
**Round 2**						
Staff should assess patients for substance use disorders at the time of admission^a^						
Clinicians	22/23 (96)	0	1/23 (4)	0	0	.01
People with LLE	5/12 (42)	5/12 (42)	1/12 (8)	0	1/12 (8)	
Patients on oxygen should not be allowed to smoke or vape in designated areas^a^						
Clinicians	12/23 (52)	7/23 (30)	0	3/23 (13)	1/23 (4)	.01
People with LLE	2/12 (17)	2/12 (17)	3/12 (25)	1/12 (8)	4/12 (33)	
Patients should be asked if they are actively using substances in the hospital once substance use is suspected^a^						
Clinicians	16/23 (70)	6/23 (26)	0	1/23 (4)	0	.04
People with LLE	4/12 (33)	4/12 (33)	2/12 (17)	0	2/12 (17)	
Toxicology testing should be performed once substance use is suspected						
Clinicians	4/23 (17)	15/23 (65)	4/23 (17)	0	0	.006
People with LLE	0	4/12 (33)	7/12 (58)	0	1/12 (8)	
Assessment of patient decision-making capacity should be performed once substance use is suspected^a^						
Clinicians	22/23 (96)	0	1/23 (4)	0	0	.008
People with LLE	5/12 (42)	2/12 (17)	2/12 (17)	1/12 (8)	2/12 (17)	
If substances are found, they should be stored in a locked space during hospitalization						
Clinicians	16/23 (70)	5/23 (22)	1/23 (4)	1/23 (4)	0	<.001
People with LLE	0	6/11 (55)	3/11 (27)	1/11 (9)	1/11 (9)	
If substance use supplies are found, they should be stored in a locked space during hospitalization^a^						
Clinicians	18/23 (78)	4/23 (17)	0	1/23 (4)	0	<.001
People with LLE	0	7/11 (64)	2/11 (18)	1/11 (9)	1/11 (9)	
The floor nursing staff should be involved in a response to suspected or confirmed in-hospital substance use^a^						
Clinicians	21/23 (91)	1/23 (4)	0	1/23 (4)	0	.03
People with LLE	5/11 (46)	3/11 (27)	2/11 (18)	1/11 (9)	0	
A multidisciplinary meeting should be held to develop a plan to manage suspected or confirmed substance use during the hospitalization^a^						
Clinicians	9/23 (39)	14/23 (61)	0	0	0	.02
People with LLE	0	9/11 (82)	1/11 (9)	0	1/11 (9)	
Hospitals should have a protocolized response to suspected inpatient substance use^a^						
Clinicians	22/23 (96)	1/23 (4)	0	0	0	.004
People with LLE	4/11 (36)	5/11 (46)	1/11 (9)	1/11 (9)	0	
**Round 3**						
The floor nursing staff should be involved in a response to suspected or confirmed in-hospital substance use^a^						
Clinicians	22/23 (96)	0	0	0	1/23 (4)	.04
People with LLE	6/12 (50)	3/12 (25)	3/12 (25)	0	0	

Abbreviation: LLE, lived or living experience.

^a^ Recommendation meets consensus criteria.

**Table 4.** Key Concepts for Consensus Recommendations to Sometimes Implement or Rarely Implement

Key concept	Frequency^a^	Illustrative example (participant)
Implementation of the recommendation must carefully weigh the trade-offs in medical care with patient autonomy	129	“Getting some sobriety from cigarettes while inpatient can be motivating for patients to continue that outpatient. With that said, I would not want a patient to leave before medically ready just because they want to smoke and aren’t allowed to.” (Clinician) “People who use drugs make decisions that make sense to them, even if others think it’s a bad decision, as they are the experts on their lives.” (Person with LLE) “Patients should have the right to decline involvement of new personnel in their care unless there is suicidal or homicidal ideation or concerns about lack of capacity (e.g., a patient with cognitive impairment who doesn’t understand instructions).” (Clinician)
Implementation of the recommendation should be considered if further intervention is still needed after evidence-based care has been optimized	27	“Medical personnel should be screened for old, outdated opinions and treat substance use disorder appropriately according to current guidelines; the hospital personnel must understand that substance use disorder is not a character flaw.” (Person with LLE) “Sometimes the nicotine replacement therapy is not maximally dosed, which is often sufficient to prevent the need for smoking. Given that some hospitals have smoke-free campuses, we should offer patients maximal NRT and other options first, and only when needed, offer designated areas for smoking. In contrast, I think vaping should be always allowed in designated areas.” (Clinician) “[Offer short-acting opioids] only if the patient desires and other methods of pain management would be [or] have proved to be insufficient. Staff should believe the patient about their pain levels and not assume they are only trying to get more medication.” (Person with LLE)
Recommendations should be implemented if feasible given local resources	56	“While ideal to have peers and addiction specialists as part of response, need to recognize that not every hospital has resources for this, so should note this in these recommendations.” (Clinician) “Potentially great if available [referring to a peer advocate]. Will not be available at most hospitals.” (Clinician) “Ideally, they [substance use supplies] would be able to be kept in a locked space, but practically, this would be pretty challenging for the hospital.” (Clinician)
Implementation of the recommendation should be done if it would have a meaningful impact on patient care	60	“If patient check-ins are increased simply to police the patient, then they should never be implemented. Patient check increased in order to support the patient—to make sure that they are doing well after using their substances, for example—then I am supportive of this policy. The check-in should always be helpful and a positive interaction for the patient.” (Person with LLE) “Depending on the substance, this may not be necessary (I would not send confirmatory testing on cocaine, for example, given the reliability of a standard urine drug screen).” (Clinician) “I think there should be flexibility for the response to include the most appropriate team members—psychiatry could be very helpful if an uncontrolled psych disorder is contributing, for example, but they wouldn’t be needed in all cases. Similarly, addiction could be involved if we can control withdrawal symptoms or cravings.” (Clinician)
Implementation of the recommendation should consider potential harms and unintended consequences (including stigma and bias)	66	“With that said, I would not want a patient to leave before medically ready just because they want to smoke and aren’t allowed to.” (Clinician) “I am not sure if a peer advocate is necessarily the best person to lead this discussion. There are times when I think it would be better for the peer advocate to focus on relationship building and to leave these discussions about ‘policy’ to other members of the team.” (Clinician) “Once, when I went to the ER for nausea and constipation, they found my fentanyl and they told me if I wanted any diagnosis and meds they would confiscate my drugs. I left. I can imagine that if I had something more serious, I would have left also. This is not good, because people are leaving without getting care.” (Person with LLE)

Abbreviations: ER, emergency room; LLE, lived or living experience; NRT, nicotine replacement therapy.

^a^ The reported frequency is the number of responses per concept.

## Discussion

Through a Delphi process involving clinicians and people with LLE, we identified consensus recommendations (Table 2) to guide patient-centered best-practice approaches to inform local responses, including hospital policies, to in-hospital substance use. The Delphi process highlighted the need for hospital-wide interventions to guide evidence-based nonstigmatizing care to people who use drugs. To our knowledge, this is the first study to develop consensus-based expert recommendations to address in-hospital substance use and guide hospital policies. This study provides a foundation for developing systems-level approaches to address in-hospital substance use and prevent harm for patients, health care workers, and the health system while improving care.

Our study’s recommendations are organized around those which should always be considered, those which should never be considered, and those which should be considered sometimes or rarely. Many of the recommendations classified as “always implement” are consistent with prior literature on hospital-based SUD care.^36,37,38^ For example, experts agreed on providing evidence-based SUD treatment, assessing or reassessing pain management and withdrawal treatment, addressing patients’ reasons for use, and addressing in-hospital substance use in a nonstigmatizing way. Despite the broad agreement on these recommendations, implementation will likely require system-level approaches, as many hospitals do not have integrated SUD care. A 2024 systematic review by Campopiano von Klimo and colleagues^12^ identified that physician reluctance to address SUD was related to lack of knowledge, skill, cognitive capacity, and lack of institutional support.^39^ Other health care workers, including nurses, also face lack of knowledge and skill and may have stigma toward people with SUD and burnout when caring for these individuals.^40,41,42^ These factors may be addressed by our experts’ nearly unanimous consensus for always implementing systems-level approaches to improve the culture of care for people with SUD.

Only a few recommendations met consensus to never implement. Most of these related to security personnel and law enforcement involvement in addressing in-hospital substance use. Law enforcement involvement can damage the relationship between the health care system and people who use drugs and can contribute to the perception of the hospital as a risk environment that complicates medical treatment and increases the risk of patient-directed discharge, rehospitalization, and death.^17^ Not involving law enforcement may require a cultural shift and institutional support to implement, as security is provided by law enforcement in some hospitals, and some hospitals may involve law enforcement when responding to in-hospital substance use.^20^ Security involvement, irrespective of being provided by law enforcement, and searches can also be damaging to patients.^18^ Of note, this study’s experts recognized that in limited occasions when there are safety concerns for patients or staff, hospital security should be involved in the in-hospital substance use response. However, people who use drugs are often stigmatized as violent or aggressive, adding complexity to identifying when safety concerns are truly present, and this will require staff education in substance use disorders and deescalation techniques.^14^

Responses to in-hospital substance use and in-hospital substance use policies may cause harm when they are not evidence-based and include punitive measures, and they may be of limited utility if health care workers are unaware of their existence.^20^ Our findings may be used to guide responses to in-hospital substance use and create or addend existing in-hospital substance use policies. A one-size-fits-all approach may not address complex issues, and our finding that experts categorized 37% of recommendations as those that should be implemented sometimes or rarely highlights the nuances they identified in effectively addressing in-hospital substance use. Recommendations should be implemented when feasible given the available resources in the hospital system, should consider the balance between providing meaningful care and supporting patient autonomy, should consider potential unintended consequences, and should maximize evidence-based interventions. For example, experts recommended selective involvement of peer advocates, psychiatrists, addiction specialists, and social workers. This may be due to concerns about personnel availability or resources across hospital systems or the desire to ensure health care workers without specific expertise in addiction are adequately trained to address in-hospital substance use. Our findings suggest that adding personnel to the response should only occur if they can improve care in a meaningful, nonstigmatizing way. Patients should have a voice in determining who those additional personnel are.

Balancing the expertise of people with LLE and clinicians adds to the nuances we elucidated in an effective response to in-hospital substance use. While there was an abundance of agreement between clinicians and people with LLE on the final recommendations, there were some statistically significant differences, including in recommendations that met consensus. People with LLE were less likely than clinicians to prioritize recommendations that limited decision-making autonomy as well as those that could lead to unintended consequences from stigmatization. These recommendations included decisions about whom to involve in the response and assessment of decision-making capacity. Clinicians were more likely than people with LLE to consider the safety and feasibility of a response within a hospital system, as evidenced by differences in responses to visitor searches, storage of substance use supplies, and smoking or vaping for patients on oxygen. When determining an approach to in-hospital substance use, both perspectives merit consideration. Based on our findings, we propose a model that incorporates both perspectives for addressing in-hospital substance use (Figure).

**Figure.**
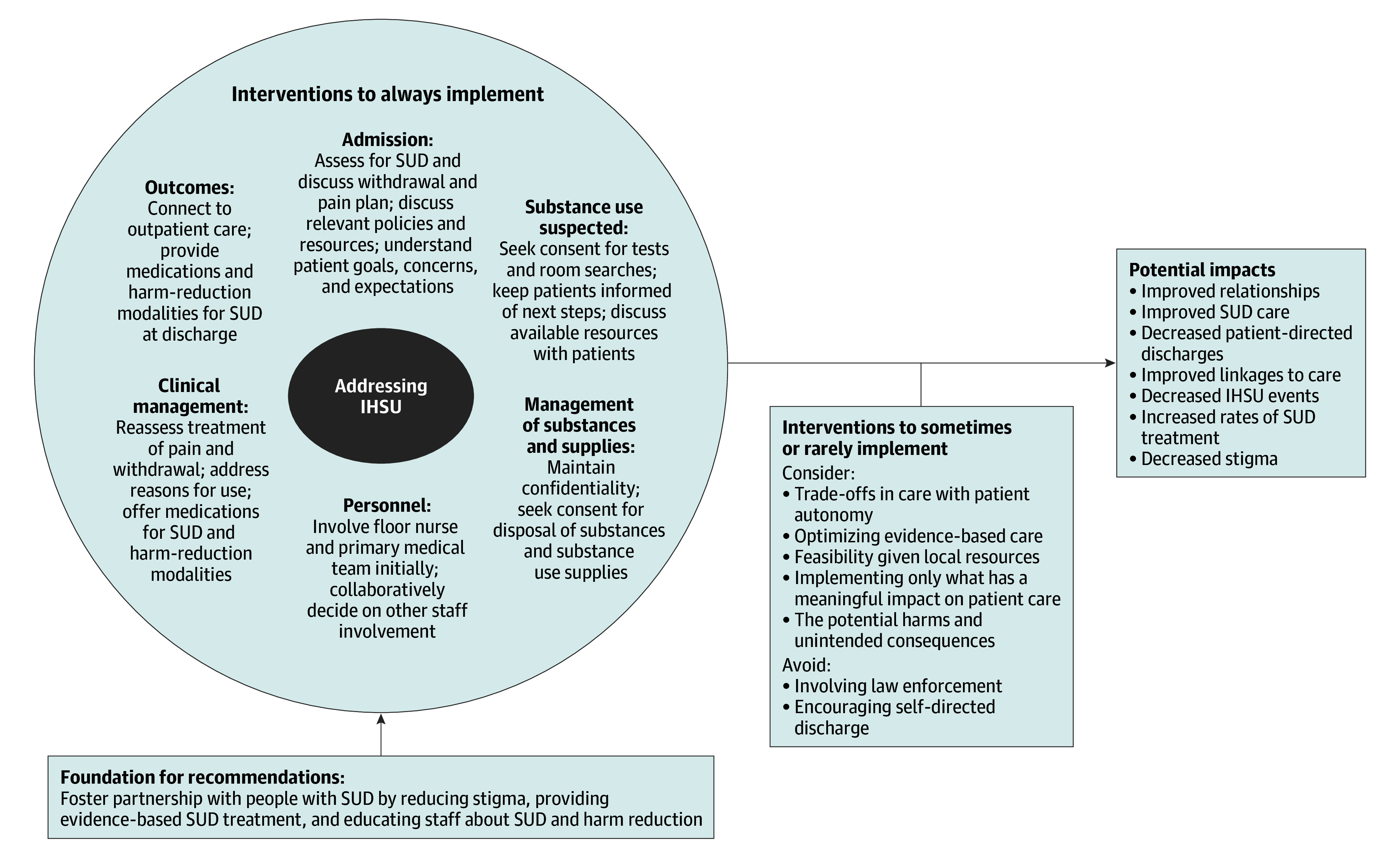
Model to Address In-Hospital Substance Use Based on the Expert Panel This model incorporates the best-practice recommendations to address in-hospital substance use. The full list of consensus recommendations is in Table 2. IHSU indicates in-hospital substance use; SUD, substance use disorder.

### Strengths and Limitations

Our study’s strengths include the consensus-based methods and inclusion of participants with SUD expertise, both hospital clinicians and people with LLE. There are also some limitations to this study. First, our recruitment focused on geographic diversity and achieving similar numbers of physicians, nonphysician clinicians, and people with LLE. Racial and ethnic minority individuals are underrepresented in this study, paralleling the lack of diversity in addiction medicine, and this may have limited the consensus recommendations.^43^ This point is important given worse outcomes among minoritized populations with SUD, including overdose, deaths, incarceration, and undertreatment.^44,45,46,47,48^ Policies and recommendations focused on people with SUD should be reviewed through an equity lens with ongoing evaluation for disproportionate harms. Second, our recruitment strategy used author group contacts, which could bias the consensus results toward uniform recommendations. However, our approach helped with study feasibility, the author group represented regional and practice diversity, and many experts were 2 or 3 degrees removed from the author group, resulting from the snowball sampling technique. Third, our recommendations are broad and do not address local regulatory nuances that may impact implementation. The feasibility of the recommendations will need to be considered by each unique hospital system. Importantly, most recommendations do not require a specialized addiction team to implement, although feasibility and implementation in diverse hospital settings are areas for further study. Fourth, specific populations of hospitalized patients who meet diagnostic criteria for SUD, such as peripartum people, were not addressed and should be thoughtfully considered when developing and implementing strategies to address in-hospital substance use given the additional harms and significance, including child protective services involvement. Fifth, our expert panel did not include hospital executive leadership, regulatory experts, hospital security directors, and floor nurses without SUD expertise. This was intentional, as we aimed to recruit participants with expertise in addiction and provide recommendations based in clinical addiction practice and lived experience. However, non-SUD experts often respond to and are impacted by in-hospital substance use and should be included in discussions of local strategies and trainings to address in-hospital substance use. Sixth, we did not include recommendations around overdose prevention centers within hospitals, as this is not currently possible in the US.^49^

## Conclusions

In this survey study, we identified recommendations that can be used to guide local approaches, including policy development, to address in-hospital substance use. In-hospital substance use among people who use drugs is common. Misguided approaches to addressing in-hospital substance use can lead to patient-directed discharges, deterioration of health care worker–patient relationships, missed opportunities to engage interested patients in SUD treatment, mistrust of the medical system, stress and anxiety for health care workers, stress on the hospital system, and poor patient outcomes. In addition to this study’s consensus recommendations, institutions should engage hospital and community partners and consider local regulations and resources when developing a policy to address in-hospital substance use in concert with other efforts to improve the care of people with SUD.

## Notes

Supplement 1.
eAppendix.
**eFigure 1.** Consensus Methodology Flowchart **eFigure 2.** Flow of Recommendations and Participation **eTable.** Recommendations Not Meeting Consensus Criteria (<80% Agreement by Experts)

Supplement 2.
Data Sharing Statement
