# Supplementary material for: Recommendations for Addressing In-Hospital Substance Use From a National Delphi Consensus Process
Source: JAMA Netw Open. 2025 Aug 25;8(8):e2528703. doi: 10.1001/jamanetworkopen.2025.28703 (PMC12379083; doi:10.1001/jamanetworkopen.2025.28703)
Supplement: Supplement 1. — eAppendix. eFigure 1. Consensus Methodology Flowchart eFigure 2. Flow of Recommendations and Participation eTable. Recommendations Not Meeting Consensus Criteria (<80% Agreement by Experts) [file jamanetwopen-e2528703-s001.pdf]

Supplementary Online Content

a

Donroe JH, Calcaterra SL, Simon C, et al. Recommendations for addressing in-hospital substance use from a national Delphi consensus process. *JAMA Netw Open*. 2025;8(8):e2528703. doi:10.1001/jamanetworkopen.2025.28703

eAppendix
eFigure 1. Consensus Methodology Flowchart
eFigure 2. Flow of Recommendations and Participation
eTable. Recommendations Not Meeting Consensus Criteria (<80% Agreement by Experts)

This supplementary material has been provided by the authors to give readers additional information about their work.

© 2025 Donroe JH et al. *JAMA Network Open*.

Inpatient Substance Use Round 2

Start of Block: Introduction

Q1 Thank for you for participating in this study. Our purpose is to develop consensus recommendations for addressing the use or suspected use of non-prescribed/non-ordered substances during an acute hospitalization.

End of Block: Introduction

Start of Block: Instructions

Instructions For the purposes of this survey, imagine your ideal hospital policy to address the use or suspected use of substances by a patient during an acute hospitalization. Examples of substances for our purposes includes any opioid (e.g., heroin, fentanyl, oxycodone), stimulant (e.g., cocaine, methamphetamine), sedative or anxiolytic (e.g., benzodiazepine, GHB) used by a patient during hospitalization but not ordered or prescribed by the patient's medical provider team.   The following survey contains recommendations that may be found within your ideal policy. For each recommendation, indicate if you believe it should "never be implemented", "rarely be implemented", "sometimes be implemented", "always be implemented", or you are "undecided". Space is provided if you feel additional recommendations should be added and if you feel the language used to describe the recommendation should be adjusted.

Q3 Do you agree to continue?

- ○ No (1)
- ○ Yes (2)

Skip To: End of Survey If Do you agree to continue? = No

© 2025 Donroe JH et al. *JAMA Network Open*.

Q4 Do you believe that hospitals should have a policy in place to address how staff respond when a patient uses or is suspected of using substances during a hospitalization?

- ○ No (1)
- ○ Yes (2)

Skip To: End of Survey If Do you believe that hospitals should have a policy in place to address how staff respond when a p... = No

End of Block: Instructions

Start of Block: Demographics

*

Q7 What is your age?

____

Q57 How do you currently describe your gender identity?

- ○ Cisgender female (1)
- ○ Cisgender male (2)
- ○ Transgender female (3)
- ○ Transgender male (7)
- ○ Genderqueer/ nonbinary (4)
- ○ Prefer not to answer (5)
- ○ Other (6)

© 2025 Donroe JH et al. *JAMA Network Open*.

Q58 How do you describe your racial/ethnic identity (Check all that apply)?

- ☐ American Indian (1)
- ☐ Alaska Native (2)
- ☐ Asian: Chinese (4)
- ☐ Asian: Filipino (5)
- ☐ Asian: Asian Indian (6)
- ☐ Asian: Vietnamese (7)
- ☐ Asian: Korean (8)
- ☐ Asian: Japanese (9)
- ☐ Asian: Other Asian (10)
- ☐ Black or African American (11)
- ☐ Hispanic, Latinx/o/a/e (12)
- ☐ Native Hawaiian (13)
- ☐ Pacific Islander: Samoan (14)
- ☐ Pacific Islander: Chamorro (15)
- ☐ Pacific Islander: Marshallese (16)
- ☐ Other Pacific Islander (17)
- ☐ White (18)
- ☐ Prefer not to answer (19)

© 2025 Donroe JH et al. *JAMA Network Open*.

☐ Other (please specify) (20)

Display this question:

If How do you describe your racial/ethnic identity (Check all that apply)? = Other (please specify)

Q59 Other racial/ethnic identity: Please describe

____

End of Block: Demographics

Start of Block: Eligibility Criteria

Q5 Participant role (check all that apply)

☐ Person with lived experience (Having a substance use disorder, but no longer using) (1)

☐ Person with living experience (Having a substance use disorder, and still using) (9)

☐ Clinician (Physician, Nurse, APP, Social Worker) (2)

End of Block: Eligibility Criteria

Start of Block: Clinician Detail

Q63 Clinician role

○ Physician (2)

○ Nurse (RN) (3)

○ Social worker (4)

○ Advanced Practice Provider (e.g. PA, NP, APRN) (5)

© 2025 Donroe JH et al. *JAMA Network Open*.

Q11 In the past year, how many weeks did you work on an inpatient addiction consult service?

- ○ <4 weeks (1)
- ○ 4-10 weeks (2)
- ○ >10-20 weeks (3)
- ○ >20 weeks (4)

Skip To: End of Survey If In the past year, how many weeks did you work on an inpatient addiction consult service? = <4 weeks

Page Break

© 2025 Donroe JH et al. *JAMA Network Open*.

Q8 What is your specialty? (check all that apply)

- ☐ Internal Medicine (1)
- ☐ Psychiatry (2)
- ☐ Psychiatry Consult Liason (8)
- ☐ Addiction Medicine (3)
- ☐ Family Medicine (7)
- ☐ Obstetrics and Gynecology (4)
- ☐ Emergency Medicine (9)
- ☐ Pediatrics (5)
- ☐ N/A (6)

Q9 Are you board certified in Addiction Medicine or Addiction Psychiatry?

- ○ No (1)
- ○ Yes (2)
- ○ N/A (3)

---

*

Q10 How many years have you worked in the field of addiction?

____

© 2025 Donroe JH et al. *JAMA Network Open*.

Q47 **The follow questions refer to the hospital you are primarily affiliated with**

Q10 Type of hospital (check all that apply)

- Academic (1)
- Federal Government (2)
- For Profit (3)
- Nonprofit (4)
- Community (5)
- Teaching (6)
- Non-Teaching (7)

Q12 Approximate size of the hospital (number of inpatient beds)

- <100 (1)
- 100-499 (2)
- 500-749 (3)
- 750-999 (4)
- >1000 (5)

© 2025 Donroe JH et al. *JAMA Network Open*.

Q13 Hospital setting

- ◯ Urban (1)
- ◯ Rural (2)
- ◯ Suburban (3)

© 2025 Donroe JH et al. *JAMA Network Open*.

Q14 Hospital location

- Alabama (1)
- Alaska (2)
- Arizona (3)
- Arkansas (4)
- California (5)
- Colorado (6)
- Connecticut (7)
- Delaware (8)
- Florida (9)
- Georgia (10)
- Hawaii (11)
- Idaho (12)
- Illinois (13)
- Indiana (14)
- Iowa (15)
- Kansas (16)
- Kentucky (17)
- Louisiana (18)
- Maine (19)
- Maryland (20)
- Massachusetts (21)

© 2025 Donroe JH et al. *JAMA Network Open*.

○ Michigan (22)

○ Minnesota (23)

○ Mississippi (24)

○ Missouri (25)

○ Montana (26)

○ Nebraska (27)

○ Nevada (28)

○ New Hampshire (29)

○ New Jersey (30)

○ New Mexico (31)

○ New York (32)

○ North Carolina (33)

○ North Dakota (34)

○ Ohio (35)

○ Oklahoma (36)

○ Oregon (37)

○ Pennsylvania (38)

○ Rhode Island (39)

○ South Carolina (40)

○ South Dakota (41)

○ Tennessee (42)

© 2025 Donroe JH et al. *JAMA Network Open*.

- Texas (43)
- Utah (44)
- Vermont (45)
- Virginia (46)
- Washington (47)
- West Virginia (48)
- Wisconsin (49)
- Wyoming (50)
- Other (51)

© 2025 Donroe JH et al. *JAMA Network Open*.

Q15 Addiction services available at or through the hospital you are primarily affiliated with- check all that apply

- ▢ Addiction Medicine consult service (1)
- ▢ Addiction Psychiatry consult service (2)
- ▢ Social Work consult (4)
- ▢ Emergency Department addiction treatment initiation (i.e. buprenorphine, naltrexone etc.) (5)
- ▢ 12 Step meetings (6)
- ▢ General Psychiatry/Consult Liason (7)
- ▢ Peer Support counselors (8)
- ▢ Patient Navigator (3)
- ▢ Syringe Exchange services (9)
- ▢ Other (10) ____
- ▢ None that I am aware of (11)

End of Block: Clinician Detail

Start of Block: PWLE Details

Q17 Have you ever been hospitalized as an adult (18 years and older)?

- ○ No (1)
- ○ Yes (2)

Skip To: End of Survey If Have you ever been hospitalized as an adult (18 years and older)? = No

© 2025 Donroe JH et al. *JAMA Network Open*.

Display this question:

If Have you ever been hospitalized as an adult (18 years and older)? = Yes

Q18 When was your most recent hospitalization?

- Within the past 1 year (1)
- Between 1 and 3 years ago (2)
- More than 3 but less than 5 years ago (3)
- 5 or more years ago (4)

Skip To: End of Survey If When was your most recent hospitalization? = 5 or more years ago

Q19 Did you use substances during your hospitalization?

- No (1)
- Yes (2)
- I prefer not to answer (3)

© 2025 Donroe JH et al. *JAMA Network Open*.

Q13 What substance use disorder(s) have you been or could you be diagnosed with? (check all that apply)

- ▢ Opioid use disorder (1)
- ▢ Alcohol use disorder (2)
- ▢ Stimulant use disorder (such as cocaine, methamphetamine) (3)
- ▢ Cannabis use disorder (4)
- ▢ Phencyclidine use disorder (5)
- ▢ Sedative use disorder (such as benzodiazepines) (6)
- ▢ Tobacco use disorder (7)
- ▢ Other (8)

Display this question:

If What substance use disorder(s) have you been or could you be diagnosed with? (check all that apply) = Other

Q14 Which other substance use disorders have you been or could you be diagnosed with?

____

Q15 Are you currently in recovery?

- ○ No (1)
- ○ Yes (2)

Display this question:

If Are you currently in recovery? = Yes

© 2025 Donroe JH et al. *JAMA Network Open*.

Q16 Approximately how long have you been in recovery?

- 0-3 months (1)
- >3-12 months (2)
- More than 12 months (3)

Q64 Do you have a role(s) advocating for people with substance use disorders?

- No, I do not have a role advocating for people with substance use disorders (9)
- Yes, I have a role(s) advocating for people with substance use disorders (6)

Display this question:

If Do you have a role(s) advocating for people with substance use disorders? = Yes, I have a role(s) advocating for people with substance use disorders

Q6 Please list your role(s) advocating for people with substance use disorders.

____

____

____

____

____

Q49 In which setting do you live?

- Urban (1)
- Rural (2)
- Suburban (3)

© 2025 Donroe JH et al. *JAMA Network Open*.

Q48 In which state do you live?

- ◯ Alabama (1)
- ◯ Alaska (2)
- ◯ Arizona (3)
- ◯ Arkansas (4)
- ◯ California (5)
- ◯ Colorado (6)
- ◯ Connecticut (7)
- ◯ Delaware (8)
- ◯ Florida (9)
- ◯ Georgia (10)
- ◯ Hawaii (11)
- ◯ Idaho (12)
- ◯ Illinois (13)
- ◯ Indiana (14)
- ◯ Iowa (15)
- ◯ Kansas (16)
- ◯ Kentucky (17)
- ◯ Louisiana (18)
- ◯ Maine (19)
- ◯ Maryland (20)
- ◯ Massachusetts (21)

© 2025 Donroe JH et al. *JAMA Network Open*.

- Michigan (22)
- Minnesota (23)
- Mississippi (24)
- Missouri (25)
- Montana (26)
- Nebraska (27)
- Nevada (28)
- New Hampshire (29)
- New Jersey (30)
- New Mexico (31)
- New York (32)
- North Carolina (33)
- North Dakota (34)
- Ohio (35)
- Oklahoma (36)
- Oregon (37)
- Pennsylvania (38)
- Rhode Island (39)
- South Carolina (40)
- South Dakota (41)
- Tennessee (42)

© 2025 Donroe JH et al. *JAMA Network Open*.

○ Texas (43)

○ Utah (44)

○ Vermont (45)

○ Virginia (46)

○ Washington (47)

○ West Virginia (48)

○ Wisconsin (49)

○ Wyoming (50)

○ Other (51)

End of Block: PWLE Details

Start of Block: General

*

Q20 Please provide your email address so that we may send you the follow up surveys:

____

*

Q47 Re-type your email address:

____

End of Block: General

Start of Block: At the time of admission

© 2025 Donroe JH et al. *JAMA Network Open*.

Q22 Recommendations for **all patients** at the time of admission:

© 2025 Donroe JH et al. *JAMA Network Open*.

	Undecided (1)	Never Implement (2)	Rarely Implement (3)	Sometimes Implement (4)	Always Implement (5)
Staff should assess patients for substance use disorders (1)	○	○	○	○	○
Staff should assess a patient's risk of withdrawal from substances (2)	○	○	○	○	○
Medication for addiction treatment should be offered if a patient meets criteria (3)	○	○	○	○	○
The patient's pain management plan should be discussed, if applicable (4)	○	○	○	○	○
The patient's withdrawal management plan should be discussed, if applicable (5)	○	○	○	○	○
Patients with a history of substance use disorder should be searched (6)	○	○	○	○	○
Patients should be offered the opportunity to discreetly dispose of substances or substance use supplies (7)	○	○	○	○	○

© 2025 Donroe JH et al. *JAMA Network Open*.

Patients should be offered the opportunity to discreetly store substances or substance use supplies with their belongings until time of discharge (8)	○	○	○	○	○
Patients should be made aware of the hospital's substance use policies (9)	○	○	○	○	○
Patients should be made aware of when their right to privacy may be superseded by the hospital’s obligation to provide safe medical care (10)	○	○	○	○	○
Patients should be made aware of why it is unsafe to use substances during a hospitalization (11)	○	○	○	○	○
Patients should be made aware of visitor expectations (12)	○	○	○	○	○

© 2025 Donroe JH et al. *JAMA Network Open*.

Patient should be asked to sign a document stating they understand the hospital policy addressing inpatient substance use (13)	○	○	○	○	○
Patients with tobacco use disorder should be offered nicotine replacement therapy (14)	○	○	○	○	○
Patients with tobacco use disorder should be allowed to smoke/vape in designated areas (15)	○	○	○	○	○

Q23 (Optional) Suggest alternative language to describe any of the above practices/outcomes. Use the space to explain your rationale for suggested changes.

____

____

____

____

____

© 2025 Donroe JH et al. *JAMA Network Open*.

Q36 (Optional) In the space provided, please provide any additional recommendations **at the time of admission** not already listed that you believe should be included in a policy addressing the use or suspected use of substances during a hospitalization:

____

____

____

____

____

End of Block: At the time of admission

Start of Block: Investigation once substance use is suspected

© 2025 Donroe JH et al. *JAMA Network Open*.

Q24 Recommendations once substance use is suspected:

© 2025 Donroe JH et al. *JAMA Network Open*.

	Undecided (1)	Never Implement (2)	Rarely Implement (3)	Sometimes Implement (4)	Always Implement (5)
Patients should be asked if they are actively using substances in the hospital (1)	○	○	○	○	○
Toxicology screening should be offered (2)	○	○	○	○	○
Patients should consent to toxicology testing (11)	○	○	○	○	○
Toxicology screening should be performed (3)	○	○	○	○	○
Screening toxicology should be followed by confirmatory testing (10)	○	○	○	○	○
Patients should be informed about why the toxicology test is being performed (4)	○	○	○	○	○
Patients should be informed about how the results will be used in their care (5)	○	○	○	○	○
Assessment of patient decision-making capacity should be performed (a clinician would evaluate the patient's ability to make their own medical decisions in that moment) (6)	○	○	○	○	○

© 2025 Donroe JH et al. *JAMA Network Open*.

Patient consent should be obtained prior to a search of room and personal belongings (7)	○	○	○	○	○
The patient's room and belongings should be searched by the clinical staff (8)	○	○	○	○	○
The patient's room and belongings should be searched by hospital security (9)	○	○	○	○	○
When available, the patient should be offered a peer advocate to assist with patient-staff interactions (12)	○	○	○	○	○

Q25 (Optional) Suggest alternative language to describe any of the above practices/outcomes. Use the space to explain your rationale for suggested changes.

____

____

____

____

____

Q51 (Optional) In the space provided, please provide any additional recommendations **once substance use is suspected** not already listed that you believe should be included in a policy addressing the use or suspected use of substances during a hospitalization:

____

© 2025 Donroe JH et al. *JAMA Network Open*.

____

____

____

____

End of Block: Investigation once substance use is suspected

Start of Block: Management of substances and substance use supplies if found

© 2025 Donroe JH et al. *JAMA Network Open*.

Q26 Recommendations on what should happen to substances and/or substance use supplies once found:

© 2025 Donroe JH et al. *JAMA Network Open*.

	Undecided (1)	Never Implement (2)	Rarely Implement (3)	Sometimes Implement (4)	Always implement (5)
Any substances found in the patient room or on their person should be taken (1)	○	○	○	○	○
Patient confidentiality should be maintained (evidence linking the patient to any illegal substances is avoided) (2)	○	○	○	○	○
Disposal of substances should occur by hospital staff (3)	○	○	○	○	○
Suspected illegal substances should be given to local law enforcement (4)	○	○	○	○	○
Substances and/or substance use supplies should be stored in a locked space during hospitalization (5)	○	○	○	○	○
Substances and/or substance use supplies should be returned to patients at time of discharge, if desired (6)	○	○	○	○	○

© 2025 Donroe JH et al. *JAMA Network Open*.

Q27 (Optional) Suggest alternative language to describe any of the above practices/outcomes. Use the space to explain your rationale for suggested changes.

____

____

____

____

____

Q52 (Optional) In the space provided, please provide any additional recommendations **on what should happen to substances and/or substance use supplies** not already listed that you believe should be included in a policy addressing the use or suspected use of substances during a hospitalization:

____

____

____

____

____

End of Block: Management of substances and substance use supplies if found

Start of Block: Personnel involved

© 2025 Donroe JH et al. *JAMA Network Open*.

Q28 Recommendation on who should be involved in a response to suspected or confirmed in-hospital substance use (**when available**):

© 2025 Donroe JH et al. *JAMA Network Open*.

	Undecided (1)	Never Implement (2)	Rarely Implement (3)	Sometimes Implement (4)	Always implement (5)
Local law enforcement should be involved (1)	○	○	○	○	○
The patient's parole/probation officer should be alerted (if applicable) (2)	○	○	○	○	○
Hospital security personnel should be involved (3)	○	○	○	○	○
The patient's primary medical/surgical team should be involved (4)	○	○	○	○	○
The floor nursing staff should be involved (5)	○	○	○	○	○
The floor or unit leadership should be involved (6)	○	○	○	○	○
A social worker should be involved (7)	○	○	○	○	○
An addiction specialist should be involved (8)	○	○	○	○	○
A psychiatry consultant or behavioral health team should be involved (9)	○	○	○	○	○
Peer advocates should be involved (10)	○	○	○	○	○

© 2025 Donroe JH et al. *JAMA Network Open*.

Patient advocates (patient relations) should be involved (11)	○	○	○	○	○
A multi-disciplinary group should be created to address inpatient substance use (12)	○	○	○	○	○

Q29 (Optional) Suggest alternative language to describe any of the above practices/outcomes. Use the space to explain your rationale for suggested changes.

____

____

____

____

____

Q53 (Optional) In the space provided, please provide any additional recommendations **on personnel involved** not already listed that you believe should be included in a policy addressing the use or suspected use of substances during a hospitalization:

____

____

____

____

____

End of Block: Personnel involved

Start of Block: Management

© 2025 Donroe JH et al. *JAMA Network Open*.

Q30 Recommendations on management once substance use during a hospitalization is suspected or confirmed:

© 2025 Donroe JH et al. *JAMA Network Open*.

	Undecided (1)	Never Implement (2)	Rarely Implement (3)	Sometimes Implement (4)	Always implement (5)
A multi-disciplinary meeting should be held to develop a plan to manage suspected or confirmed substance use during the hospitalization (1)	○	○	○	○	○
The patient's pain management plan should be assessed or reassessed (2)	○	○	○	○	○
The patient's withdrawal management plan should be assessed or reassessed (3)	○	○	○	○	○
The patient's triggers for use (craving, stress, anxiety…) should be assessed or reassessed (4)	○	○	○	○	○
Medications for symptom management should be offered (5)	○	○	○	○	○
Medication for addiction treatment should be offered if patient meets criteria (6)	○	○	○	○	○
Addiction medication dosage should be increased if appropriate and patient desires (7)	○	○	○	○	○

© 2025 Donroe JH et al. *JAMA Network Open*.

Available harm reduction modalities should be discussed (8)	○	○	○	○	○
Short acting oral opioid agonists should be converted to liquid form (9)	○	○	○	○	○
Patients should be offered psychosocial treatment groups during the hospitalization (if available) (10)	○	○	○	○	○
The patient should be reminded of the hospital's policy, including next steps should substance use continue (11)	○	○	○	○	○
The patient should sign an agreement expressing understanding of the hospital's policy for inpatient substance use (12)	○	○	○	○	○

Q31 (Optional) Suggest alternative language to describe any of the above practices/outcomes. Use the space to explain your rationale for suggested changes.

____

____

____

____

____

© 2025 Donroe JH et al. *JAMA Network Open*.

Q54 (Optional) In the space provided, please provide any additional recommendations **on management** not already listed that you believe should be included in a policy addressing the use or suspected use of substances during a hospitalization:

____

____

____

____

____

End of Block: Management

Start of Block: Documentation

© 2025 Donroe JH et al. *JAMA Network Open*.

Q32 Recommendations on clinical documentation:

	Undecided (1)	Never Implement (2)	Rarely Implement (3)	Sometimes Implement (4)	Always implement (5)
Conversations about policies addressing the use of substances should be documented in the patient's medical record (1)	○	○	○	○	○
Investigation into suspected substance use should be documented in the patient's medical record (2)	○	○	○	○	○
Reasons for suspecting substance use during the hospitalization should be documented in the patient's medical record (3)	○	○	○	○	○
The management of suspected or confirmed substance use in the hospital should be documented in the patient's medical record (4)	○	○	○	○	○
An alert should be added to the patient's chart identifying them as someone who used substances during their hospitalization (5)	○	○	○	○	○

© 2025 Donroe JH et al. *JAMA Network Open*.

Q33 (Optional) Suggest alternative language to describe any of the above practices/outcomes. Use the space to explain your rationale for suggested changes.

____

____

____

____

____

Q55 (Optional) In the space provided, please provide any additional recommendations **on documentation** not already listed that you believe should be included in a policy addressing the use or suspected use of substances during a hospitalization:

____

____

____

____

____

End of Block: Documentation

Start of Block: Outcomes from the response to substance use in the hospital

© 2025 Donroe JH et al. *JAMA Network Open*.

Q34 Recommendation on the expected outcomes from a response to substance use in the hospital:

© 2025 Donroe JH et al. *JAMA Network Open*.

	Undecided (1)	Never Implement (2)	Rarely Implement (3)	Sometimes Implement (4)	Always implement (5)
The treating physician can discharge patients from the hospital if the patient refuses to comply with hospital policy (1)	○	○	○	○	○
Patients should be instructed to self direct their discharge ("discharge against medical advice") from the hospital if they refuse to comply with hospital policy (2)	○	○	○	○	○
Patients who self direct their discharge should be connected to outpatient addiction care, if desired (3)	○	○	○	○	○
Patients who self direct their discharge should be prescribed medications for addiction treatment, if desired (4)	○	○	○	○	○

© 2025 Donroe JH et al. *JAMA Network Open*.

Patients who self direct their discharge should be provided with harm reduction modalities (i.e. naloxone, syringes, skin prep, etc.), if desired (5)	○	○	○	○	○
Patients should lose visitation privileges (6)	○	○	○	○	○
If visitation privileges are maintained, all visitors should be searched prior to visitation (7)	○	○	○	○	○
Patients should be restricted to their room (8)	○	○	○	○	○
Patient's room should be relocated to a more easily observable location (9)	○	○	○	○	○
A behavioral agreement between the patient and hospital staff should be drafted (10)	○	○	○	○	○

© 2025 Donroe JH et al. *JAMA Network Open*.

Furniture with drawers and any personal containers where syringes or drugs or alcohol could be stored should be removed from the patient’s room (11)	○	○	○	○	○
Restriction of pass privileges (ability to leave the medical ward) should be implemented (12)	○	○	○	○	○
A safety attendant (in person or remotely) should be ordered to monitor the patient (13)	○	○	○	○	○
A pathway to re-establish lost privileges during the hospitalization should be available (14)	○	○	○	○	○

Q35 (Optional) Suggest alternative language to describe any of the above practices/outcomes. Use the space to explain your rationale for suggested changes.

____

____

____

____

© 2025 Donroe JH et al. *JAMA Network Open*.

Q56 (Optional) In the space provided, please provide any additional recommendations **on expected outcomes** not already listed that you believe should be included in a policy addressing the use or suspected use of substances during a hospitalization:

End of Block: Outcomes from the response to substance use in the hospital

Start of Block: Optional

Q37 (Optional) In the space provided, please provide any additional comments you feel relevant to guiding policy to address the use or suspected use of substances during an acute hospitalization:

Q46 (Optional) Please suggest expert clinicians, including email address, to participate in this study.

© 2025 Donroe JH et al. *JAMA Network Open*.

Q50 (Optional) Please suggest expert persons with lived/living experience, including email address, to participate in this study.

End of Block: Optional

© 2025 Donroe JH et al. *JAMA Network Open*.

eFigure 1: Consensus Methodology Flowchart

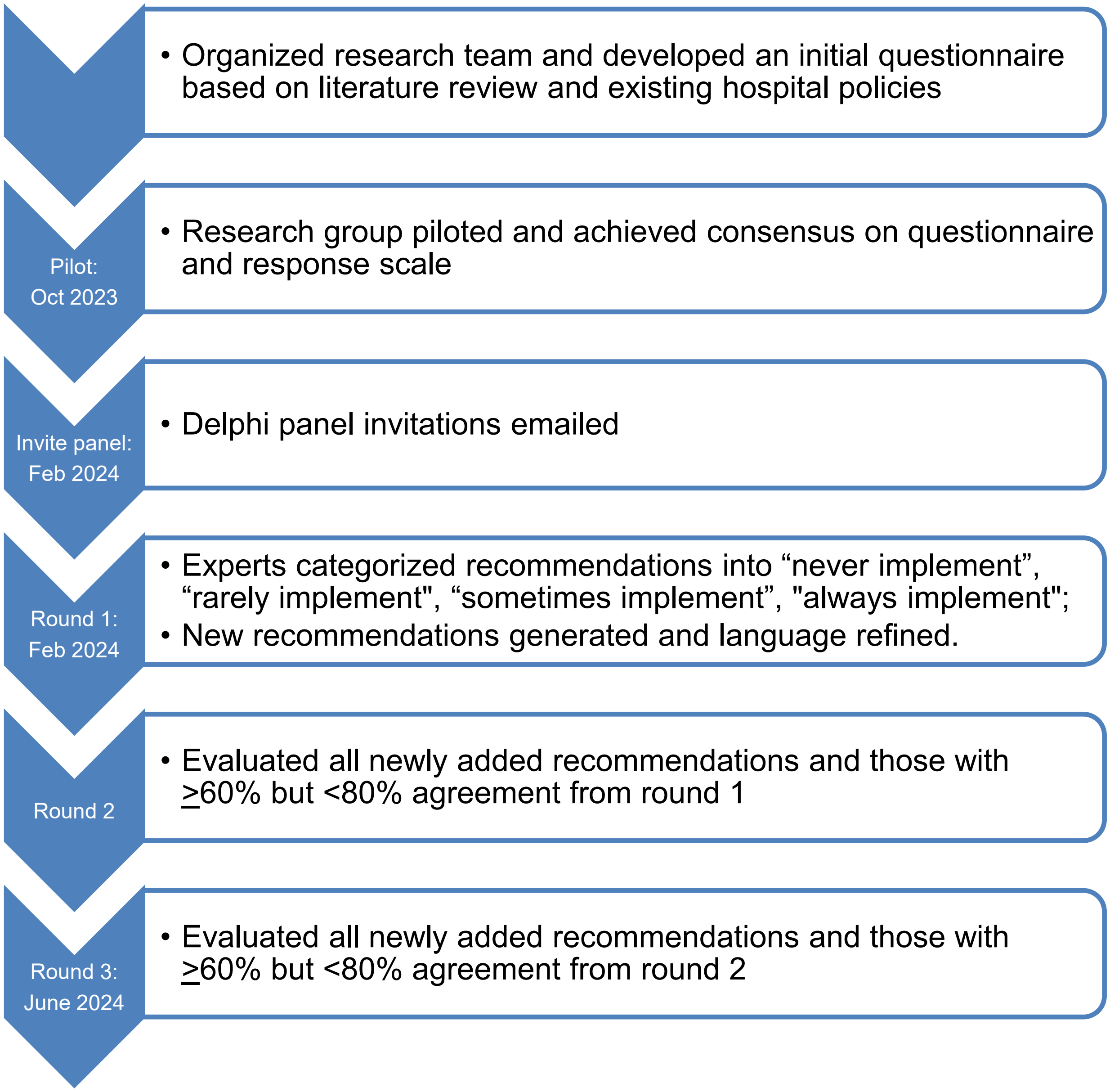

© 2025 Donroe JH et al. *JAMA Network Open*.

eFigure 2: Flow of recommendations and participation

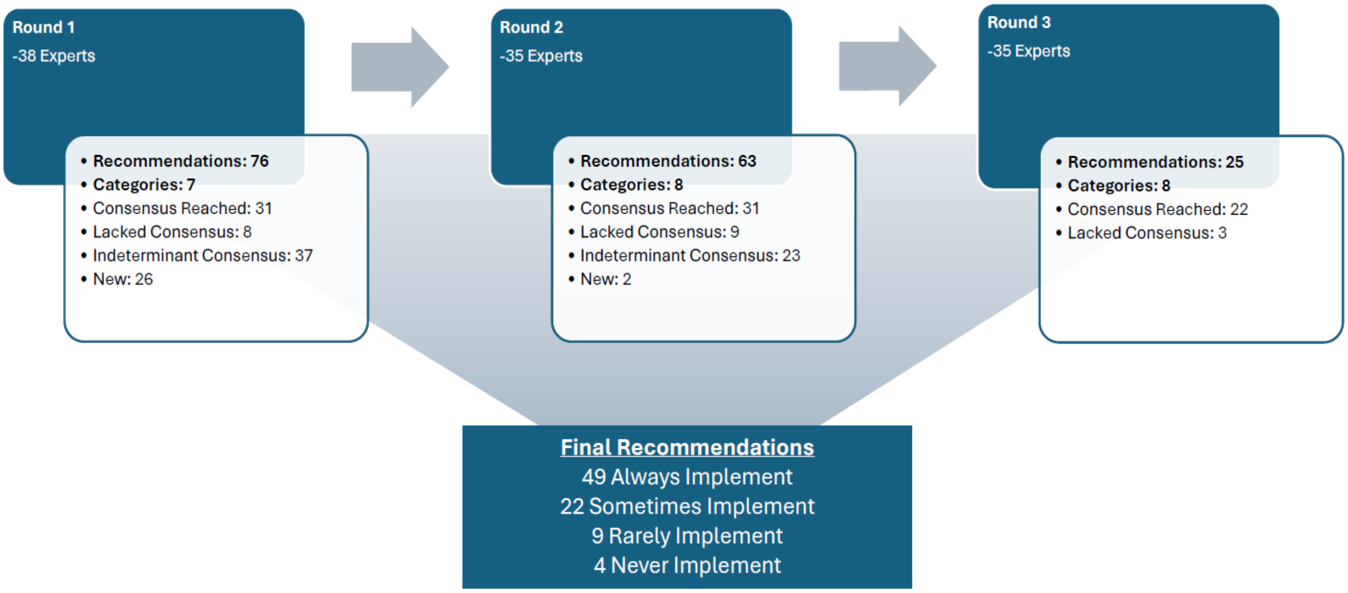

© 2025 Donroe JH et al. *JAMA Network Open*.

eTable: Recommendations not meeting consensus criteria (<80% agreement by experts)

**Recommendation**	**%**				
	**A**	**S**	**R**	**N**	**U**
**All patients at the time of admission**					
1. Patient should be asked to sign a document stating they understand the hospital policy addressing inpatient substance use	21	32	11	13	24
**Investigation once substance use is suspected**					
2. The patient's room/ belongings should be searched by security if there is a safety concern for clinical staff or patients	11	40	31	11	7
3. Toxicology screening should be offered	29	46	20	-	6
4. Toxicology screening should be performed	11	54	32	-	3
5. The patient's room and belongings should be searched by the clinical staff	3	20	31	46	-
6. If patient does not consent, staff can search a patient's room or belongings if considered necessary	11	23	43	20	3
**Management of substances and substance use supplies if found**					
7. Any substances found in the patient room or on their person should be taken	29	26	11	19	16
8. Disposal of substances should occur by hospital staff	24	34	24	11	7
9. Substances should be stored in a locked space during hospitalization	47	32	12	6	3
**Personnel involved**					
10. The floor or unit leadership should be involved	27	50	18	3	2
**Clinical management**					
11. Short acting oral opioid agonists should be converted to liquid form	13	45	26	3	13
12. The patient should sign an agreement expressing understanding of the hospital's policy for inpatient substance use	29	18	16	16	21
**Documentation**					
13. Investigation into suspected substance use should be documented in the patient's medical record	16	21	24	32	7
14. Reasons for suspecting substance use during the hospitalization should be documented in the patient's medical record	21	29	18	24	8
15. Conversations about policies addressing the use of substances should be documented in the patient's medical record	65	15	3	18	-

© 2025 Donroe JH et al. *JAMA Network Open*.

16. The management of suspected or confirmed substance use in the hospital should be documented in the patient’s medical record	44	29	12	9	6
**Outcomes from the response to inpatient substance use**					
17. The treating physician can discharge patients from the hospital if the patient refuses to comply with hospital policy	11	26	13	40	11
18. A behavioral agreement between the patient and hospital staff should be drafted	18	44	21	15	2
19. Patient visitors should be restricted	-	29	50	15	6
20. Patient's room should be relocated to a more easily observable location	6	21	47	24	3

Always (A), Sometimes (S), Rarely (R), Never (N), Undecided (U)

© 2025 Donroe JH et al. *JAMA Network Open*.
