# Supplementary material for: Recommendations for Addressing In-Hospital Substance Use From a National Delphi Consensus Process
Source: JAMA Netw Open. 2025 Aug 25;8(8):e2528703. doi: 10.1001/jamanetworkopen.2025.28703 (PMC12379083; doi:10.1001/jamanetworkopen.2025.28703)
Supplement: Supplement 2. — Data Sharing Statement [file jamanetwopen-e2528703-s002.pdf]

Data Sharing Statement

Donroe. Recommendations for Addressing In-Hospital Substance Use From a National Delphi Consensus Process. *JAMA Netw Open*. Published August 25, 2025. doi:10.1001/jamanetworkopen.2025.28703

Data
Data available: No

Additional Information
Explanation for why data not available: Data are not shared because we inquire about personal experience with substances use, and it would be a violation of trust with those who participated.
